# The Prognostic Importance of CD20^+^ B lymphocytes in Colorectal Cancer and the Relation to Other Immune Cell subsets

**DOI:** 10.1038/s41598-019-56441-8

**Published:** 2019-12-27

**Authors:** Sofia Edin, Tuomas Kaprio, Jaana Hagström, Pär Larsson, Harri Mustonen, Camilla Böckelman, Karin Strigård, Ulf Gunnarsson, Caj Haglund, Richard Palmqvist

**Affiliations:** 1 Department of Medical Biosciences/Pathology, Umeå University, Umeå, Sweden; 2 Department of Surgical and Perioperative Sciences/Surgery, Umeå University, Umeå, Sweden; 3 Department of Surgery, University of Helsinki and Helsinki University Hospital, Helsinki, Finland; 4 Translational Cancer Medicine Research Program, Faculty of Medicine, University of Helsinki, Helsinki, Finland; 5 Department of Pathology, University of Helsinki and Helsinki University Hospital, Helsinki, Finland

**Keywords:** Colorectal cancer, Adaptive immunity

## Abstract

The anti-tumour immune response is critical to patient prognosis in colorectal cancer (CRC). The aim of this study was to investigate infiltration of B lymphocytes into CRC tumours, and their clinical relevance, prognostic value and relation to other immune cell subsets. We used multiplexed immunohistochemistry and multispectral imaging to assay the amount of infiltrating CD20^+^ B lymphocytes along with infiltration of CD8^+^ cytotoxic T cells, FOXP3^+^ T regulatory cells, CD68^+^ macrophages and CD66b^+^ neutrophils, in 316 archival CRC tissue specimens. A higher density of infiltrating CD20^+^ B lymphocytes was associated with tumours of the right colon (*P* = 0.025) and of lower stages (*P* = 0.009). Furthermore, patients whose tumours were highly infiltrated by CD20^+^ B lymphocytes had a significantly improved disease-specific survival (HR = 0.45, 95% CI 0.28–0.73, *P* = 0.001), which remained significant in multivariable analysis. CD20^+^ B lymphocytes were highly and positively associated with CD8^+^ T lymphocytes (*P* < 0.001), and part of the prognostic role was found to be a cooperative effect between these lymphocyte subsets. Our results support a favourable prognostic value of tumour-infiltrating CD20^+^ B lymphocytes in CRC. Furthermore, a cooperative prognostic effect between CD20^+^ B lymphocytes and CD8^+^ T lymphocytes is suggested.

## Introduction

Despite medical advances, CRC remains one of the most deadly cancers worldwide^1^. Curative treatment is based on surgical resection, but still almost half of the patients will die of their disease due to tumour metastasis. Immune infiltration has been proven to be of powerful prognostic value in CRC^2^. In the era of immunotherapy, a more detailed understanding of how the immune response is organised to counteract tumour growth and spread, may lead to important prognostic clues and new targets for therapy.

The adaptive immune response is orchestrated by antigen-specific T and B lymphocytes. T lymphocytes are known combaters in anti-tumour immunity and can inhibit tumour growth by direct killing (cytotoxic T lymphocytes)^3^. The prognostic importance of infiltrating subsets of T lymphocytes in CRC has been widely accepted, and subsequently led to a joint task force to introduce the Immunoscore, based on immunohistochemical (IHC) evaluation of T cell markers, into clinical practice^2,4^. The role of infiltrating B lymphocytes is less explored and in matters of prognostic importance consensus has yet to be reached^5^. In addition to the adaptive immune response, cells of innate immunity are found at the tumour site. Macrophage infiltration has been linked to an improved prognosis in CRC^6^, while the prognostic importance of neutrophils is still uncertain^7–10^.

Both the intratumoural localisation and functional orientation of immune cells have been shown to carry prognostic information. For instance, the strongest prognostic value of the cytotoxic T lymphocytes in CRC is found within the tumour epithelium^11^, while most other immune cell subsets mainly reside in the tumour stromal compartment. Infiltration of regulatory T lymphocytes is somewhat surprisingly also associated with an improved prognosis in CRC, but a higher ratio of CD8^+^ to FOXP3^+^ cells does appear to improve prognosis^11–13^. Similar trends have been seen when comparing the ratio of tumour infiltrating M1 to M2 subsets of macrophages^14^. In the Th1/Th2 paradigm, the activity of the cytotoxic T cells is supported by the Th1 lineage and M1 macrophages, while in contrast regulatory T lymphocytes, B lymphocytes and M2 macrophages are more closely related to the tumour promoting Th2 response^15^. However, coordinated T and B lymphocyte responses are well established in both autoimmunity and allograft rejection^16,17^. In addition, small lymphoid organizations that contain both T and B lymphocytes - called tertiary lymphoid structures (TLS) - are detected in tumours and linked to a potent lymphocyte response and a good prognosis, suggesting that the B lymphocytes may collaborate with T lymphocytes in anti-tumour immunity^18^. Towards the goal of developing more efficient therapies, understanding the role of B lymphocytes in the immune response to CRC is critical.

In this study, we have used multiplexed IHC and multispectral imaging to analyse the degree of infiltration of five different immune cells belonging to both the adaptive (CD20^+^ B lymphocytes, CD8^+^ cytotoxic T lymphocytes, and FOXP3^+^ T regulatory cells,) and the innate (CD68^+^ macrophages and CD66b^+^ neutrophils) immune system, in CRC tissue specimens. By this we could study the individual clinical relevance and prognostic importance of B lymphocytes, but also the interrelation with other immune cell subsets and their combined prognostic value.

## Results

### Analyses of the distribution of infiltrating immune cells in CRC tumour tissues

**Figure 1:**
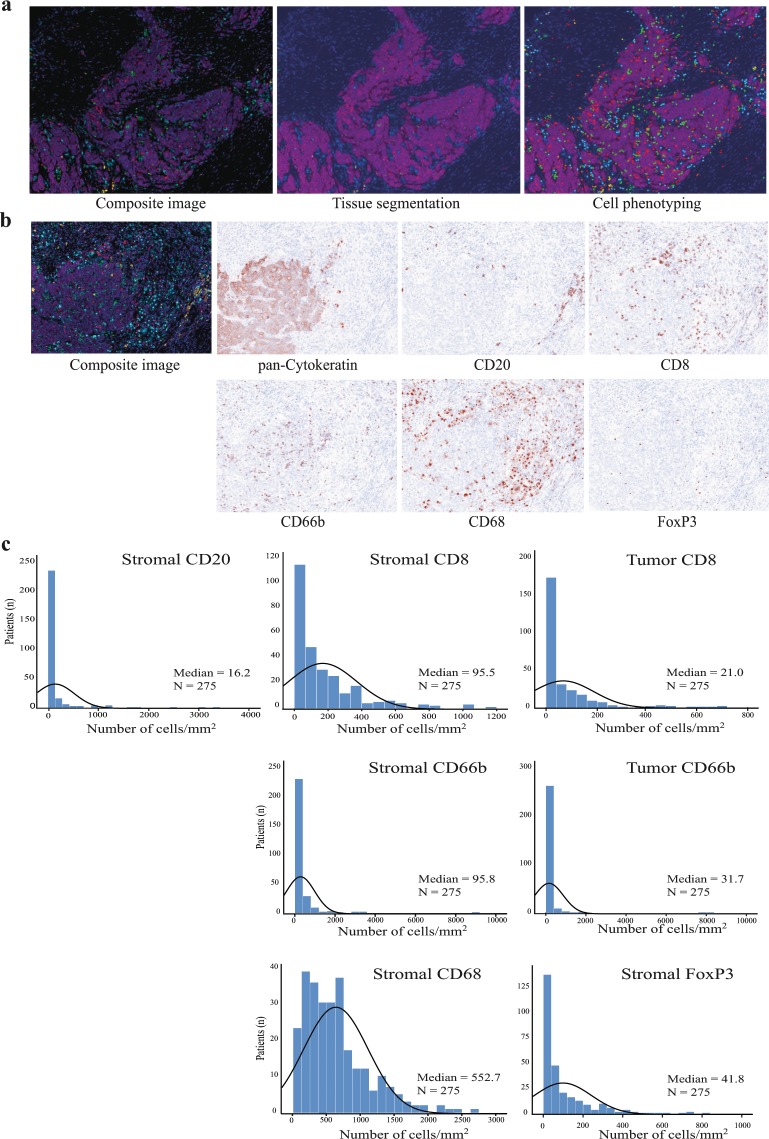
Multispectral imaging of immune cell infiltrates in CRC tissues. (**a**) A high-magnification area (20x) of a representative multiplexed IHC stained composite image after imaging and spectral unmixing (left panel), after tissue segmentation into tumour (magenta) and stromal (blue) compartments (middle panel), and after cell phenotyping (right panel). The following colors were used to identify the different markers; pan-Cytokeratin (magenta), CD20 (yellow), CD8 (red), CD66b (green), CD68 (cyan), FoxP3 (orange), and DAPI (blue). (**b**) Illustrates the staining pattern of the individual markers on a high-resolution area of a representative TMA core, after spectral unmixing, using the pathology view tool. (**c**) Histograms displaying the distribution (number of cells per mm^2^) of infiltrating immune cell subsets in the cohort of 275 CRC patients. In addition, the normal curve and the median number of infiltrating cells is shown.

We analysed a cohort of 316 CRC patients for local infiltration of immune cell subsets using multiplexed IHC staining and multispectral image analysis. Immune cell subsets were identified by sequential staining of CD66b (neutrophils), CD8 (cytotoxic T lymphocytes), CD20 (B lymphocytes), CD68 (macrophages) and FoxP3 (T regulatory cells). Pan-Cytokeratin was used to identify tumour tissue, and DAPI was used for nuclear counterstaining. Spectral unmixing resulted in a composite image displaying the different immune markers (Fig. 1a). Machine-learning algorithms were trained for tissue segmentation into different tumour compartments (tumour tissue, stromal tissue and no tissue), cell segmentation and cell phenotyping (Fig. 1a) to identify each of the different immune markers (Fig. 1b). After exclusions, immune data from 275 patients was collected and presented as number of cells per mm^2^ (Fig. 1c). The exclusion criteria are described in detail in the materials and methods section. In brief, 36 patients were excluded due to lack of immune data from both TMA cores. For 66 of the 275 study patients, immune data was collected from one TMA core. CD8 and CD66b were evident and scored in both tumour and stromal compartments, while data from the remaining immune cell markers was collected solely from the stromal compartment. Overall, macrophages were found to be the most prominent immune cells infiltrating CRC stromal tissues, followed by neutrophils, cytotoxic T lymphocytes, T regulatory cells and B lymphocytes (Fig. 1c). Immune infiltration has been vigorously studied in CRC, however the role of B lymphocytes in immune regulation and prognosis in CRC is still unclear. We therefore turned our interest to the CD20^+^ B lymphocyte population.

### Relation of infiltrating CD20^+^ cells to clinical characteristics and other immune cell subsets in CRC patients

Tumour infiltrating immune cell subsets were divided into groups of high or low infiltration based on the median as a cut-off, and analysed for the associations to patient’s clinical characteristics. CD20^+^ cells were more often highly infiltrating tumours of the right colon (Table 1; *P* = 0.025) and tumours of lower stages (Table 1; *P* = 0.009). This pattern closely follows that seen for most other immune cell subsets (Supplementary Table S2), with the exception of FoxP3, which was not significantly associated with tumour localisation, and CD66b, which was not significantly associated with tumour stage. Preoperative radiotherapy was administered to 33% of rectal cancers, and irradiated rectal cancers were significantly associated with a reduced infiltration of CD20^+^ cells (Table 1), as well as CD8^+^ cells and CD66b^+^ cells (Supplementary Table S2). We found no significant associations with age or sex (Table 1). In line with a similar clinical distribution, infiltrating CD20^+^ cells significantly correlated to the presence of all other immune cell subsets analysed, with the strongest correlation to CD8^+^ cells (Table 2).

**Table 1:** Associations to clinicopathological characteristics.

	*n*	Stromal CD20		*P*-value
		Low	High	
**Frequency, n (%)**	275	138 (50.2)	137 (49.8)	
**Mean number of cells/mm**^**2**^ **(±s.d.)**		4.4 (±4.8)	255.1 (±516.3)	
**Age, n (%)**				
≤59	74	36 (48.6)	38 (51.4)	0.964/0.703*
60–69	73	37 (50.7)	36 (49.3)	
70–79	83	41 (49.4)	42 (50.6)	
≥80	45	24 (53.3)	21 (46.7)	
**Sex, n (%)**				
Women	132	64 (48.5)	68 (51.5)	0.589
Men	143	74 (51.7)	69 (48.3)	
**Localisation, n (%)**				
Right-sided colon	73	29 (39.7)	44 (60.3)	0.080/0.025*
Left-sided colon	55	27 (49.1)	28 (50.9)	
Rectum	147	82 (55.8)	65 (44.2)	
**Stage, n (%)**				
I	55	20 (36.4)	35 (63.6)	0.009/0.009*
II	77	41 (53.2)	36 (46.8)	
III	95	44 (46.3)	51 (53.7)	
IV	48	33 (68.8)	15 (31.3)	
**Preoperative radiotherapy**^**†**^**, n (%)**				
No	227	101 (44.5)	126 (55.5)	<0.001
Yes	48	37 (77.1)	11 (22.9)	

χ^2^ tests were used for categorical variables. *Exact linear-by-linear association test was used to test for linear relationship between variables. ^†^Preoperative radiation therapy in rectal cancers only.

**Table 2:** The correlation of infiltration of CD20 positive cells in the stromal compartment to infiltration of other immune cell markers.

Immune marker	CD20	CD20
	*r*_*s*_	*P-*value
Stromal CD8	0.568	<0.001
Tumour CD8	0.403	<0.001
Stromal CD66b	0.234	<0.001
Tumour CD66b	0.184	0.002
Stromal CD68	0.219	<0.001
Stromal FOXP3	0.237	<0.001

r_s_, Spearmans rank correlation coefficient. Analyses were performed using continuous values for number of cells/mm^2^ (n = 275).

### The prognostic importance of infiltrating CD20^+^ cells in CRC patients

**Figure 2:**
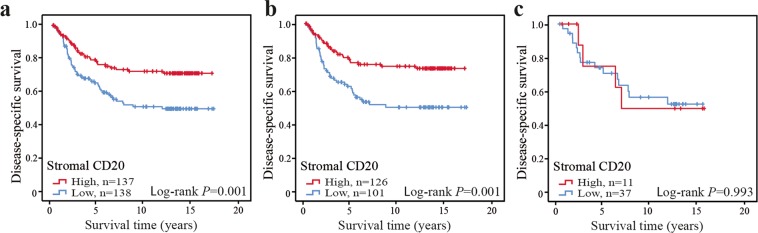
Disease-specific survival in CRC according to infiltration of CD20^+^ cells. High or low infiltration of stromal CD20 in CRC cases (**a**), in non-irradiated CRC cases (**b**), and in irradiated rectal cancers (**c**). Shown are Kaplan-Meier plots. Log-rank tests were used to calculate *P*-values.

We next investigated the relation of infiltrating CD20^+^ cells to disease-specific survival in patients with CRC. Patients with tumours highly infiltrated by CD20^+^ cells were found to have an improved prognosis compared to patients with tumours poorly infiltrated by CD20 (Fig. 2a). Since radiotherapy was associated with reduced immune infiltration, we stratified patients according to preoperative radiotherapy. CD20^+^ B cells were found to have prognostic value in patients with non-irradiated CRC tumours (Fig. 2b), but not in patients with irradiated rectal cancers (Fig. 2c). A trend of reduced prognostic value in irradiated patients was seen also for the remaining immune cell subsets (Supplementary Table S3). We therefore decided to focus the continued prognostic studies on the patients with non-irradiated CRCs.

According to our results, patients with tumours highly infiltrated by CD20^+^ cells had a significantly improved disease-specific survival (Table 3; HR = 0.45, 95% CI 0.28–0.73, *P* = 0.001), which remained significant in a multivariable Cox regression model including age, localisation, stage, and stratified by sex (Table 3; HR 0.60, 95% CI 0.37‒0.99; *P = *0.045). The prognostic value of CD8, CD68 and FoxP3, previously demonstrated in literature, was confirmed by our study (Table 3), validating the method used. Our results additionally support a favourable prognostic role of CD66b^+^ cells. For CD8^+^ cells and CD68^+^ cells, as well as CD66b^+^ cells infiltrating the tumour compartment, the prognostic value remained in multivariable analysis (Table 3).

**Table 3:** Cox regression analyses of infiltrating immune cells in predicting survival of CRC patients.

Immune marker	univariable			multivariable^a^		
	HR	95% CI	*P-*value	HR	95% CI	*P*-value
Stromal CD20	0.45	0.28–0.73	0.001	0.60	0.37–0.99	0.045
Stromal CD8	0.32	0.20–0.53	<0.001	0.42	0.25–0.72	0.002
Tumour CD8	0.31	0.19–0.51	<0.001	0.54	0.33–0.90	0.018
Stromal CD66b	0.60	0.38–0.96	0.033	0.73	0.45–1.17	0.189
Tumour CD66b	0.47	0.29–0.75	0.002	0.56	0.34–0.92	0.022
Stromal CD68	0.38	0.23–0.62	<0.001	0.55	0.31–0.96	0.034
Stromal FoxP3	0.31	0.19–0.51	<0.001	0.75	0.43–1.31	0.318

^a^The Cox regression multivariable models included one categorical immune parameter, age, localisation and stage, and were stratified by sex (n = 217). Abbreviations: HR, hazard ratio; CI, confidence interval.

### The prognostic importance of infiltrating CD20^+^ cells in relation to other immune cell subsets

**Figure 3:**
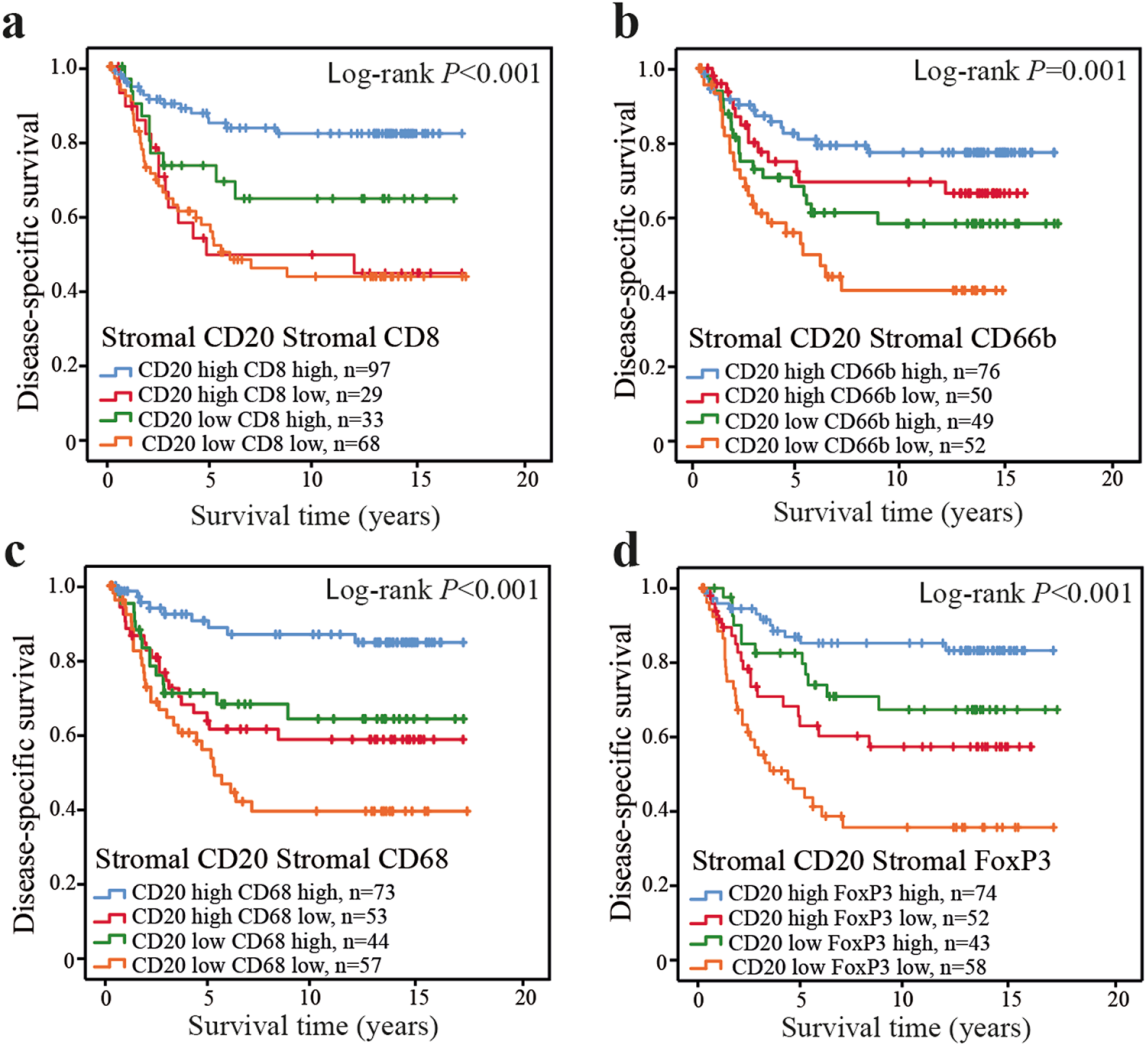
Prognostic relation of CD20^+^ cells to different immune cell subsets in CRC. Cases scored for subgroups of CD20 high or low together with; (**a**) CD8 high or low; (**b**) CD66b high or low; (**c**) CD68 high or low; and (**d**) FoxP3 high or low, in the tumour stromal compartment as indicated. Shown are Kaplan-Meier plots. Log-rank tests were used to calculate *P*-values.

We next analysed the prognostic importance of CD20^+^ cells in relation to the other investigated immune cell subsets. Since the strongest correlation of infiltrating CD20^+^ cells was found to CD8^+^ cells (Table 2), we chose primarily to focus on this interrelationship. To analyse the prognostic importance of CD20^+^ cells in relation to infiltrating CD8^+^ cells, we compared the prognostic value of groups of CD20 (high or low) to that of CD8 (high or low) in the stromal compartment. The best prognosis was found in patients whose tumours were highly infiltrated by CD8^+^ cells (Table 4, Fig. 3a). However, patients with tumours highly infiltrated by both CD8 and CD20 had a slightly improved prognosis compared to patients with tumours highly infiltrated by CD8 but poorly infiltrated by CD20 (Table 4; *P* = 0.043; Fig. 3a), suggesting that CD20^+^ cells may aid in the CD8^+^ cell mediated anti-tumour response. In fact, in patients with tumours poorly infiltrated by CD8^+^ cells, the prognostic role of CD20 infiltration was diminished. We found similar relations of CD20 infiltration also to stromal CD66b, CD68 and FoxP3 infiltration (Table 4; Fig. 3b–d). However, in patients with tumours poorly infiltrated by CD66b, CD68 and FoxP3, CD20 infiltration did still appear to have a prognostic advantage (Fig. 3b–d).

**Table 4:** Prognostic relations of immune cell subsets in CRC.

Immune marker		*n*	univariable			multivariable^a^		
			HR	95% CI	*P-*value	HR	95% CI	*P-*value
Stromal CD20/Stromal CD8	High/High (ref)	97	1.00	—	**<0.001**	1.00	—	**0.007**
	High/Low (1)	29	3.83	1.85–7.95	<0.001	3.36	1.54–7.34	0.002
	Low/High (2)	33	2.28	1.03–5.08	0.043	2.05	0.91–4.61	0.084
	Low/Low (3)	68	4.06	2.20–7.48	<0.001	2.87	1.49–5.51	0.002
Stromal CD20/Stromal CD66b	High/High (ref)	76	1.00	—	**0.002**	1.00	—	**0.157**
	High/Low (1)	50	1.55	0.75–3.21	0.240	1.54	0.73–3.24	0.259
	Low/High (2)	49	2.11	1.07–4.15	0.031	1.86	0.92–3.78	0.086
	Low/Low (3)	52	3.37	1.76–6.43	<0.001	2.13	1.09–4.14	0.027
Stromal CD20/Stromal CD68	High/High (ref)	73	1.00	—	**<0.001**	1.00	—	**0.018**
	High/Low (1)	53	3.45	1.57–7.58	0.002	3.27	1.41–7.59	0.006
	Low/High (2)	44	2.96	1.28–6.83	0.011	3.32	1.40–7.87	0.006
	Low/Low (3)	57	5.59	2.64–11.82	<0.001	3.38	1.49–7.67	0.004
Stromal CD20/Stromal FoxP3	High/High (ref)	74	1.00	—	**<0.001**	1.00	—	**0.194**
	High/Low (1)	52	3.10	1.46–6.57	0.003	1.34	0.60–3.02	0.476
	Low/High (2)	43	2.03	0.90–4.61	0.089	1.74	0.74–4.08	0.201
	Low/Low (3)	58	5.80	2.91–11.58	<0.001	2.11	0.99–4.50	0.053

^a^The Cox regression multivariable models included one categorical immune parameter, age, localization and stage, and were stratified by sex. Abbreviations: HR, hazard ratio; CI, confidence interval.

## Discussion

In this study, we have investigated the prognostic importance of CD20^+^ B lymphocytes in CRC and their relation to other immune cell subsets. Patients with tumours highly infiltrated by CD20^+^ B lymphocytes were found to have an independent prognostic advantage. We further found that the infiltration of CD20^+^ B lymphocytes was highly correlated to infiltration by other immune cell subsets, and that the mere part of the prognostic role of CD20^+^ B lymphocytes likely is mediated through a cooperative effect with the cytotoxic T lymphocytes.

We used multiplexed IHC and multispectral imaging to score five different tumour-infiltrating immune cells in CRC using well-established markers (CD8^+^ cytotoxic T lymphocytes, FOXP3^+^ T regulatory cells, CD20^+^ B lymphocytes, CD68^+^ macrophages, and CD66b^+^ neutrophils). CD20 is expressed throughout the different stages of B cell development, but is down-regulated upon differentiation into plasma cells^19^. CD20 is thus a marker for naive B cells, germinal center B cells, and memory B cells. The multiplex IHC method is advantageous for studies of interrelationships between different immune cell subsets, since it allows immunostaining of multiple markers within one image. Multiplex IHC and multispectral imaging has been validated as a good method to study tumour immune infiltration in two independent studies, showing reliable data and a good overlap with conventional IHC staining and evaluation^20,21^. The method is not optimal for use on whole tissue sections for a large patient cohort, so instead the study was performed using TMA. This together with the chosen digital approach has the disadvantage of not being able to assess the often heterogenic distribution of immune cells and to avoid e.g. necrotic areas. These problems were partly controlled for by manually inspecting each scanned image and when necessary excluding parts or whole images. Furthermore, tumour immune infiltration was calculated as a mean value from two individual TMA cores for most patients. An advantage of using a digital approach is, however, to overcome observer variability. A weakness of this study is that a large percentage of cases are rectal cancers, and that part of these have received preoperative radiotherapy, which may impact immune cell infiltration. Both reduced and enhanced immune responses are shown in response to irradiation^11,22–24^. For this reason, irradiated rectal cancers were excluded from the survival analyses. However, the effect of irradiation on immune infiltration in rectal cancer and the relation to prognosis is interesting, and a topic for further investigation. The well-established prognostic value of the different T lymphocyte subsets^4,11,13^ and macrophages^6,25^, as previously reported in literature using conventional IHC, could be identified also in this study, further strengthening the validity of the method used.

Only few studies have explored the prognostic role of B lymphocytes in CRC. The favourable patient prognosis seen in our cohort with a high number of tumour-infiltrating CD20^+^ B lymphocytes is well in line with a previous study by Berntsson *et al*.^26^, where they in a relatively large cohort found an independent favourable prognostic role of infiltrating CD20^+^ B lymphocytes in CRC primary tumours. In their study, they used a scoring method where they manually counted tumour infiltrating CD20^+^ cells, and analysed their prognostic value by diving them into groups of high and low tumour infiltration. In the study by Berntsson *et al*. they simultaneously showed that also infiltration of CD138^+^ plasma cells was linked to an improved prognosis. They did not however perform a combined analysis of B and T lymphocyte infiltrates. Two more studies have addressed the prognostic role of CD20^+^ B lymphocytes in CRC and found a neutral or negative prognostic role^27,28^. These were smaller studies using a semi-quantitative scale of infiltration, which may contribute to these differences. Our study reinforces a positive prognostic role of B lymphocytes in primary CRC. Infiltration by CD20^+^ B lymphocytes in metastatic CRC has been explored, and also here a beneficial impact on prognosis has been found^29,30^.

We further addressed the prognostic relation of infiltrating CD20^+^ B lymphocytes with the other immune cell subsets. A high infiltration of CD20^+^ B lymphocytes was found to slightly increase the prognostic effect of CD8^+^ cytotoxic T lymphocytes, while having no prognostic effect on patients with tumours poorly infiltrated by CD8^+^ cytotoxic T lymphocytes. A finding suggesting a cooperative prognostic effect between these lymphocyte subsets, which to our knowledge has not previously been shown in primary CRC. The combined prognostic value of CD8^+^ and CD20^+^ lymphocytes has been previously addressed in one study on metastatic CRC, where a combined additive effect was shown when both immune cell subsets were present^30^. Similarly, combined effects by T and B lymphocyte infiltration has been suggested for ovarian cancer, hepatocellular carcinoma, and pancreatic ductal adenocarcinoma^31–33^. Our study showed additive prognostic effects of CD20^+^ B lymphocytes also for CD66b^+^ neutrophils, CD68^+^ macrophages and FoxP3^+^ T regulatory cells, which may indicate interrelationships also between these immune cell subtypes. However, here the prognostic effect of CD20 was not entirely dependent on infiltration by these other immune cell subsets. Further studies are needed to evaluate these possible interactions.

There are several ways by which T and B lymphocytes may theoretically interact in anti-tumour immunity. B lymphocytes do produce cytokines that could support the T cell response. They may also act as antigen-presenting cells to T cells, and produce antibodies directed against tumour antigens. B lymphocyte-derived antibodies have been shown to recognize tumour antigens in CRC^34^, as well as e.g. lung and breast cancer^35,36^. Interestingly, the B lymphocytes were often, but not always, found in lymphoid-like follicles denoted TLS, and the presence of TLS have been linked to an improved prognosis in many cancers including CRC^18^. In CRC, a strong co-localisation of CD20 and Ki67 was additionally found within TLS, suggestive of B lymphocyte proliferation and tumour reactivity^37^. On the other hand, there are mouse data that instead hint towards a potential negative effect of tumour-infiltrating B cells in CRC^38^. At present, the role of B lymphocytes in the anti-tumour immune response and as a clinical parameter to predict outcome remains unclear. Future studies, taking into account also the phenotype and function of different B lymphocyte subsets, are needed. Further understanding of the interactions within the anti-tumour immune response may provide prognostic clues and additional tools for immunotherapy.

In conclusion, the results of our study support a positive prognostic role of tumour-infiltrating CD20^+^ B lymphocytes in CRC patients. Furthermore, a cooperative prognostic effect between CD20^+^ B lymphocytes and cytotoxic T lymphocytes is suggested.

## Materials and Methods

### Patient cohort and TMAs

The study population consisted of 316 CRC patients operated on in the years 1998–2003 in the Department of Surgery, Helsinki University Hospital. Actual survival data was provided by the Finnish Population Register Center, and cause of death was specified by Statistics Finland. The median age at diagnosis was 69 years. The study was approved by the Surgical Ethics Committee of Helsinki University Hospital (Dnro HUS 226/E6/06, extension TMK02 §66 17.4.2013) and in accordance with relevant guidelines and regulations. The National Supervisory Authority of Welfare and Health gave us permission to use tissue samples without the individual informed consent of the patients in this retrospective study (Valvira Dnro 10041/06). For construction of tissue microarray (TMA) blocks an experienced gastropathologist annotated representative tumour areas on H&E slides. The TMA blocks were constructed using a TMA Grand Master 3D instrument (Histech Ltd Budapest, Hungary) by punching 1 mm cores from archived formalin-fixed and paraffin-embedded (FFPE) tissue samples. The TMA used for this study included two 1 mm cores taken within the tumour mass, one more central and one more peripheral to avoid systematic heterogeneity

### Multiplexed immunohistochemical (IHC) staining

Multiplexed IHC staining was modified from the manufacturer´s instructions to the Opal^TM^ 7 Solid Tumour Immunology Kit (PerkinElmer, Waltham, MA, USA), but optimized for colorectal FFPE TMA tissue sections. In brief, tissue TMA slides were sequentially stained using antibodies against CD66b, CD8, CD20, FoxP3, CD68 and pan-Cytokeratin. The CD4 antibody used in the kit was exchanged by CD66b (clone 80H3, LsBio, Seattle, WA, USA). The CD8 antibody in the kit was exchanged by CD8 (clone 144b, DAKO). Concentrations of antibodies and Opal dyes were adjusted (according to Supplementary Table S1) so that the signal intensity of each marker would allow exposure times of 30–200 ms and a signal range of 5–30 ms. Slides were mounted using Prolong Diamond Antifade Mountant (ThermoFisher, Waltham MA, USA).

### Multispectral imaging

Imaging was performed using the VECTRA 3 Quantitative Pathology Imaging System (PerkinElmer). All standard epi-flourescent filters were used; DAPI, FITC, CY3, Texas Red, and CY5. Whole slide scans were acquired using x10 magnification. The Phenochart software (PerkinElmer) was subsequently used to mark TMA cores from the whole slide scans for subsequent multispectral imaging using x20 magnification. A spectral library was collected by single stainings of colorectal FFPE tissue sections with the CD20 antibody and the individual Opal dyes and subsequent imaging. An unstained sample was used as autofluorescence control and utilized together with the spectral library for spectral unmixing in the inForm software (PerkinElmer). Composite images were compared to single stained slides and inspected for crosstalk and interference.

### Image analysis and data collection

Images were quantified using the inForm software in two steps. Firstly, 15 TMA cores representing the heterogeneous nature of CRC, were selected to train machine-learning algorithms for tissue segmentation, cell segmentation and cell phenotyping, that were later applied on the whole TMA cohort. The software was first trained to segment tissue by manually annotating tumour tissue, stromal tissue, and no tissue, according to manufacturer´s recommendations. Cell segmentation was based on the nuclear DAPI stain, but assisted using nuclear FoxP3, and membrane CD8, CD66b, CD20 and CD68 staining. For classification of cell phenotypes, the software was trained by manually annotating 34–72 cells identified by each marker. Each scanned image was examined by one observer under the supervision of an experienced gastropathologist. Exclusion criteria for whole TMA cores were; loss of large part or whole core (n = 33), lack of tumour or stromal tissue (n = 32), bad quality of staining (e.g. weak pan-Cytokeratin stain) (n = 42), and heavy necrosis (n = 24). In total, this lead to the exclusion of 5 patients due to large part or whole cores lost, 3 patients due to lack of tumour or stromal tissues, 10 patients due to bad quality of staining, 5 patients due to heavy necrosis, and 13 patients were excluded for a mixture of the above described exclusion criteria. Four of the remaining patients had duplicate TMA sets, and for these patients the immune infiltration was calculated from all available TMA cores. One patient was excluded due to lack of clinical information. After exclusions, 275 patients remained in the study, of which 209 had immune data from two or more cores, and 66 had immune data from one TMA core.

We inspected each TMA core for areas of disinterest, which were manually drawn and subtracted from the image. Exclusion areas included; cellular debris, mucus, large vessels, areas of necrosis, large lymphoid aggregates and normal epithelial tissue. After exclusion of cores and regions, cell segmentation summary data was collected for each TMA core, and converted to number of individual cell types per mm^2^ (tumour or stromal compartment). For patients with data on two TMA cores, an average number of cells per mm^2^ was calculated from the total number of cells divided by the total tissue area. Infiltrating immune cells, as identified by the different markers, were further divided into groups of high and low infiltration by the median number of infiltrating cells.

### Statistics

Statistical analyses were performed using PASW Statistics 25 (SPSS Inc., Chicago, IL, USA). The χ2 test was used for cross-tabulations and the linear-by-linear association test for linear relationships. Correlations between continuous variables were analysed using the Spearman´s rank correlation test. Disease-specific survival was estimated using Kaplan-Meier survival analysis, and comparisons of differences in outcome between groups were analysed using the log-rank test. Multivariable survival analyses were performed using Cox proportional hazard models. Testing of the Cox model assumption of constant hazard ratios over time involved the inclusion of a time-dependent covariate separately for each testable variable. There was weak indication that gender did not follow the assumption, therefore, in multivariable analyses, a stratified analysis for gender was performed. Interaction terms were considered, but no significant interaction was found after the Bonferroni correction for multiple testing. *P* < 0.05 was considered statistically significant.

## Supplementary information

Supplementary Information

## Data Availability

The datasets analysed during the current study are available from the corresponding author on reasonable request.
