# Supplementary material for: The Prognostic Importance of CD20+ B lymphocytes in Colorectal Cancer and the Relation to Other Immune Cell subsets
Source: Sci Rep. 2019 Dec 27;9:19997. doi: 10.1038/s41598-019-56441-8 (PMC6934737; doi:10.1038/s41598-019-56441-8)
Supplement: Supplementary file 1 — Supplementary Information [file 41598_2019_56441_MOESM1_ESM.pdf]

Supplementary Information

“The prognostic importance of CD20^{+} B lymphocytes in colorectal cancer and the relation to other immune cell subsets”

Sofia Edin, Tuomas Kaprio, Jaana Hagström, Pär Larsson, Harri Mustonen, Camilla Böckelman, Karin Strigård, Ulf Gunnarsson, Caj Haglund, and Richard Palmqvist.

Supplementary Table S1. Concentration of antibodies and Opal dyes.

Antigen	Final concentration (μg/ml)	Opal Dye	Opal dilution
CD66b	2.5	520	1/50
CD20	5	540	1/200
CD8	0.1	570	1/100
FoxP3	0.3	620	1/100
CD68	0.2	650	1/150
CK	3.6	690	1/50

Supplementary Table S2. Associations to clinicopathological characteristics

	Stromal CD8			Tumor CD8			Stromal CD66b			Tumor CD66b			Stromal CD68			Stromal FoxP3		
	Low	High	*P* -value	Low	High	*P* -value	Low	High	*P* -value	Low	High	*P* -value	Low	High	*P* -value	Low	High	*P* -value
**Frequency, n (%)**	138 (50.2)	137 (49.8)		138 (50.2)	137 (49.8)		138 (50.2)	137 (49.8)		138 (50.2)	137 (49.8)		138 (50.2)	137 (49.8)		138 (50.2)	137 (49.8)	
**Mean number of cells/mm^2 (±s.d.)**	36.2 (±27.3)	306.7 (±212.2)		6.0 (±6.1)	134.5 (±145.1)		31.9 (±26.9)	550.4 (±933.2)		10.1 (±9.6)	311.7 (±990.4)		284.5 (±144.7)	989.0 (±432.8)		11.5 (±12.5)	187.4 (±161.5)	
**Age, n (%)**			0.863/0.514*			0.643/0.619*			0.899 / 0.790*			0.271/0.078*			0.790/0.790*			0.276/0.061*
≤59	38 (51.4)	36 (48.6)		34 (45.9)	40 (54.1)		35 (47.3)	39 (52.7)		30 (40.5)	44 (59.5)		38 (51.4)	36 (48.6)		31 (41.9)	43 (58.1)	
60-69	38 (52.1)	35 (47.9)		37 (50.7)	36 (49.3)		39 (53.4)	34 (46.6)		38 (52.1)	35 (47.9)		33 (45.2)	40 (54.8)		37 (50.7)	36 (49.3)	
70-79	42 (50.6)	41 (49.4)		46 (55.4)	37 (44.6)		41 (49.4)	42 (50.6)		45 (54.2)	38 (45.8)		44 (53.0)	39 (47.0)		43 (51.8)	40 (48.2)	
≥80	20 (44.4)	25 (55.6)		21 (46.7)	24 (53.3)		23 (51.1)	22 (48.9)		25 (55.6)	20 (44.4)		23 (51.1)	22 (48.9)		27 (60.0)	18 (40.0)	
**Sex, n (%)**			0.765			0.434			0.047			0.132			0.434			0.206
Women	65 (49.2)	67 (50.8)		63 (47.7)	69 (52.3)		58 (43.9)	74 (56.1)		60 (45.5)	72 (54.5)		63 (47.7)	69 (52.3)		61 (46.2)	71 (53.8)	
Men	73 (51.0)	70 (49.0)		75 (52.4)	68 (47.6)		80 (55.9)	63 (44.1)		78 (54.5)	65 (45.5)		75 (52.4)	68 (47.6)		77 (53.8)	66 (46.2)	
**Localization, n (%)**			<0.001/<0.001*			0.011/0.003*			0.051/0.017*			0.138/0.211*			0.001/<0.001*			0.102/0.267*
Right-sided colon	21 (28.8)	52 (71.2)		26 (35.6)	47 (64.4)		28 (38.4)	45 (61.6)		30 (41.1)	43 (58.9)		23 (31.5)	50 (68.5)		30 (41.1)	43 (58.9)	
Left-sided colon	25 (45.5)	30 (54.5)		28 (50.9)	27 (49.1)		28 (50.9)	27 (49.1)		32 (58.2)	23 (41.8)		30 (54.5)	25 (45.5)		33 (60.0)	22 (40.0)	
Rectum	92 (62.6)	55 (37.4)		84 (57.1)	63 (42.9)		82 (55.8)	65 (44.2)		76 (51.7)	71 (48.3)		85 (57.8)	62 (42.2)		75 (51.0)	72 (49.0)	
**Stage, n (%)**			0.001/<0.001*			<0.001/<0.001*			0.406/0.157*			0.068/0.058*			0.004/0.001*			<0.001/<0.001*
I	16 (29.1)	39 (70.9)		16 (29.1)	39 (70.9)		22 (40.0)	33 (60.0)		22 (40.0)	33 (60.0)		21 (38.2)	34 (61.8)		12 (21.8)	43 (78.2)	
II	35 (45.5)	42 (54.5)		36 (46.8)	41 (53.2)		40 (51.9)	37 (48.1)		41 (53.2)	36 (46.8)		36 (46.8)	41 (53.2)		36 (46.8)	41 (53.2)	
III	56 (58.9)	39 (41.1)		52 (54.7)	43 (45.3)		50 (52.6)	45 (47.4)		44 (46.3)	51 (53.7)		46 (48.4)	49 (51.6)		51 (53.7)	44 (46.3)	
IV	31 (64.6)	17 (35.4)		34 (70.8)	14 (29.2)		26 (54.2)	22 (45.8)		31 (64.6)	17 (35.4)		35 (72.9)	13 (27.1)		39 (81.3)	9 (18.8)	
**Preoperative radiotherapy, n (%)**			<0.001			<0.001			<0.001			0.978			0.214			0.214
No	97 (42.7)	130 (57.3)		101 (44.5)	126 (55.5)		102 (44.9)	125 (55.1)		114 (50.2)	113 (49.8)		110 (48.5)	117 (51.5)		110 (48.5)	117 (51.5)	
Yes	41 (85.4)	7 (14.6)		37 (77.1)	11 (22.9)		36 (75.0)	12 (25.0)		24 (50.0)	24 (50.0)		28 (58.3)	20 (41.7)		28 (58.3)	20 (41.7)	

χ^2 tests were used for categorical variables. *Exact linear-by-linear association test was used to test for linear relationship between variables. †Preoperative radiation therapy in rectal cancers only.

Supplementary Table S3. Cox regression analyses of infiltrating immune cells in predicting survival of patients with irradiated rectal cancers

	univariable		
**Immune marker**	**HR**	**95% CI**	***P*-value**
Stromal CD20	1.01	0.33 - 3.03	0.993
Stromal CD8	0.35	0.05 - 2.63	0.307
Tumor CD8	0.15	0.02 - 1.09	0.061
Stromal CD66b	2.22	0.87 - 5.67	0.097
Tumor CD66b	1.66	0.65 - 4.21	0.289
Stromal CD68	0.42	0.16 - 1.11	0.080
Stromal FoxP3	0.69	0.27 - 1.77	0.440

Abbreviations: HR, hazard ratio; CI, confidence interval.
